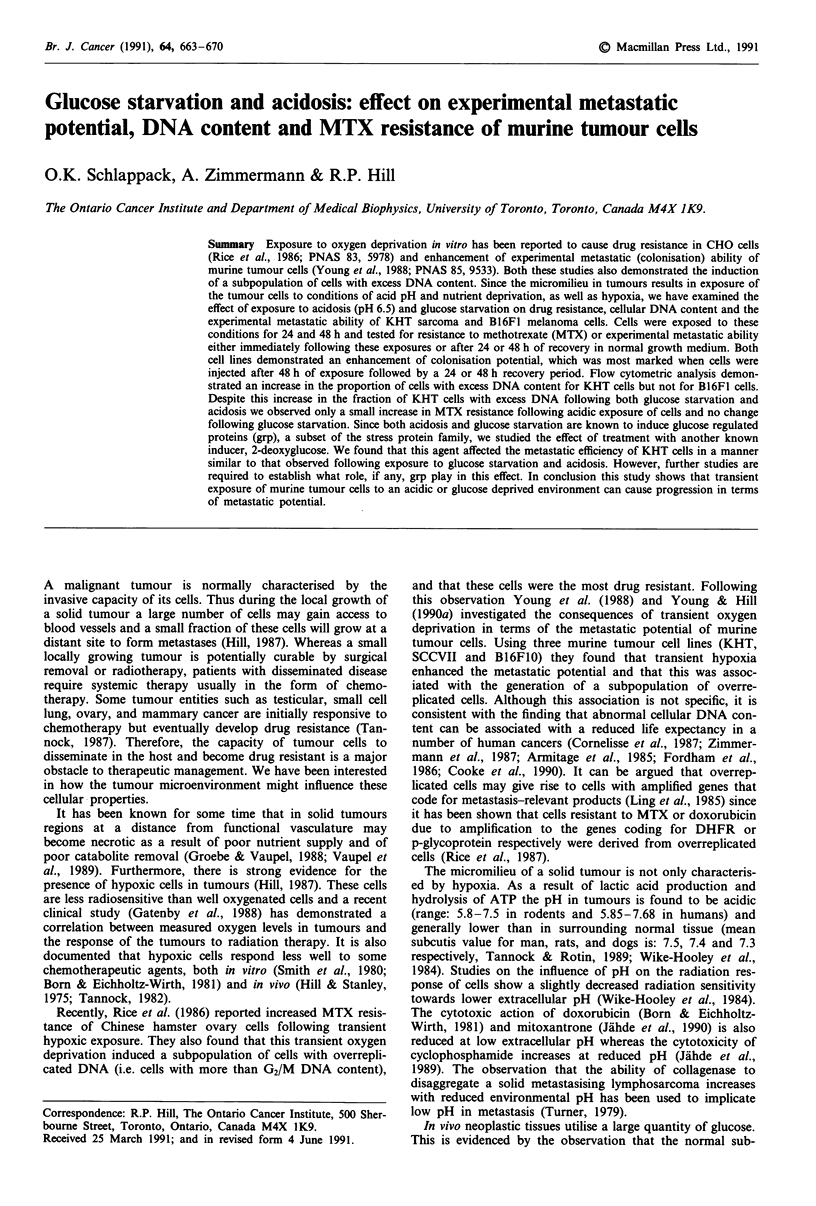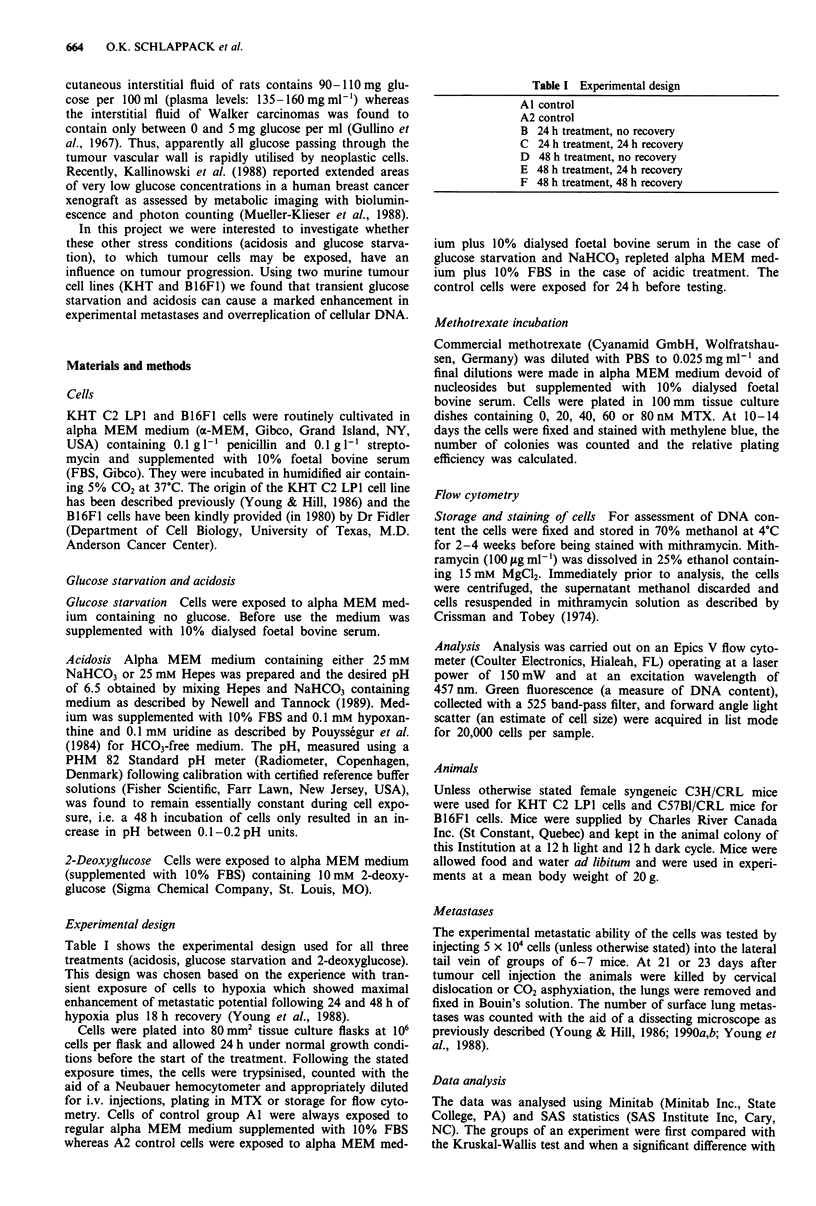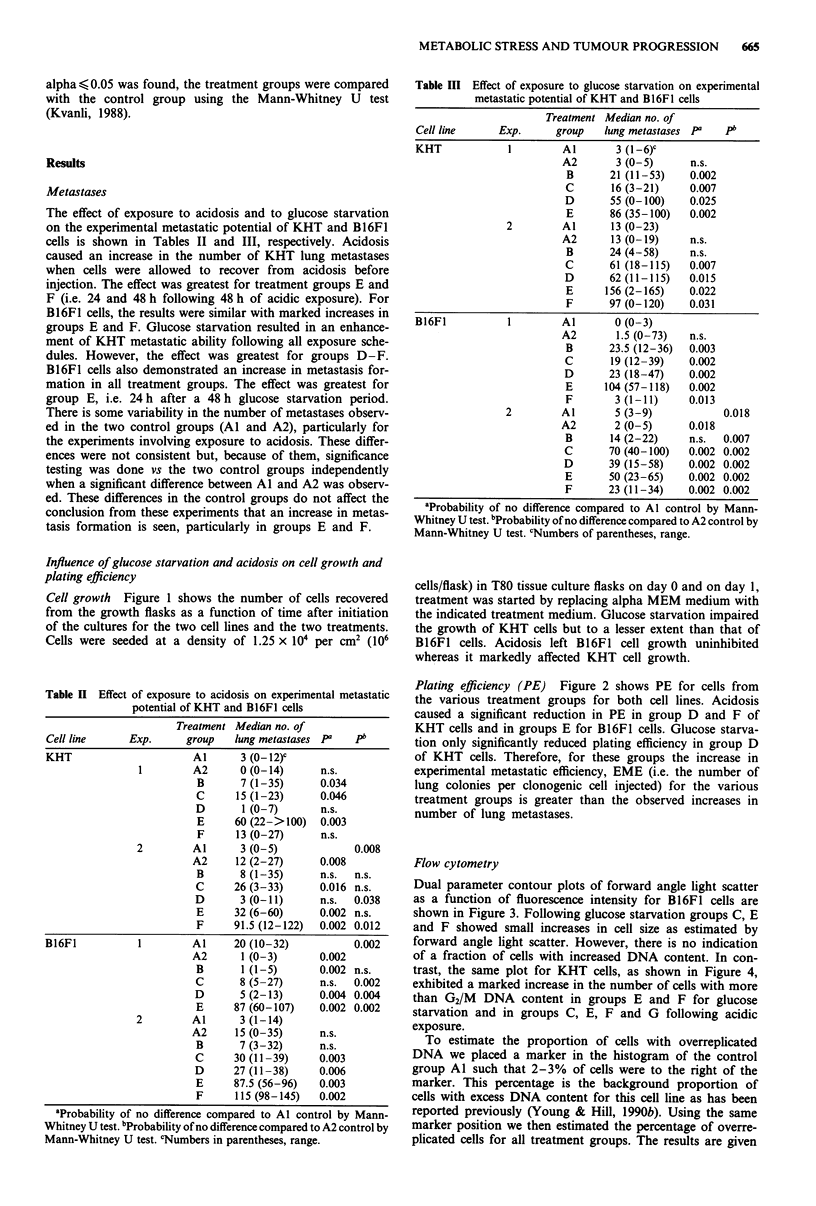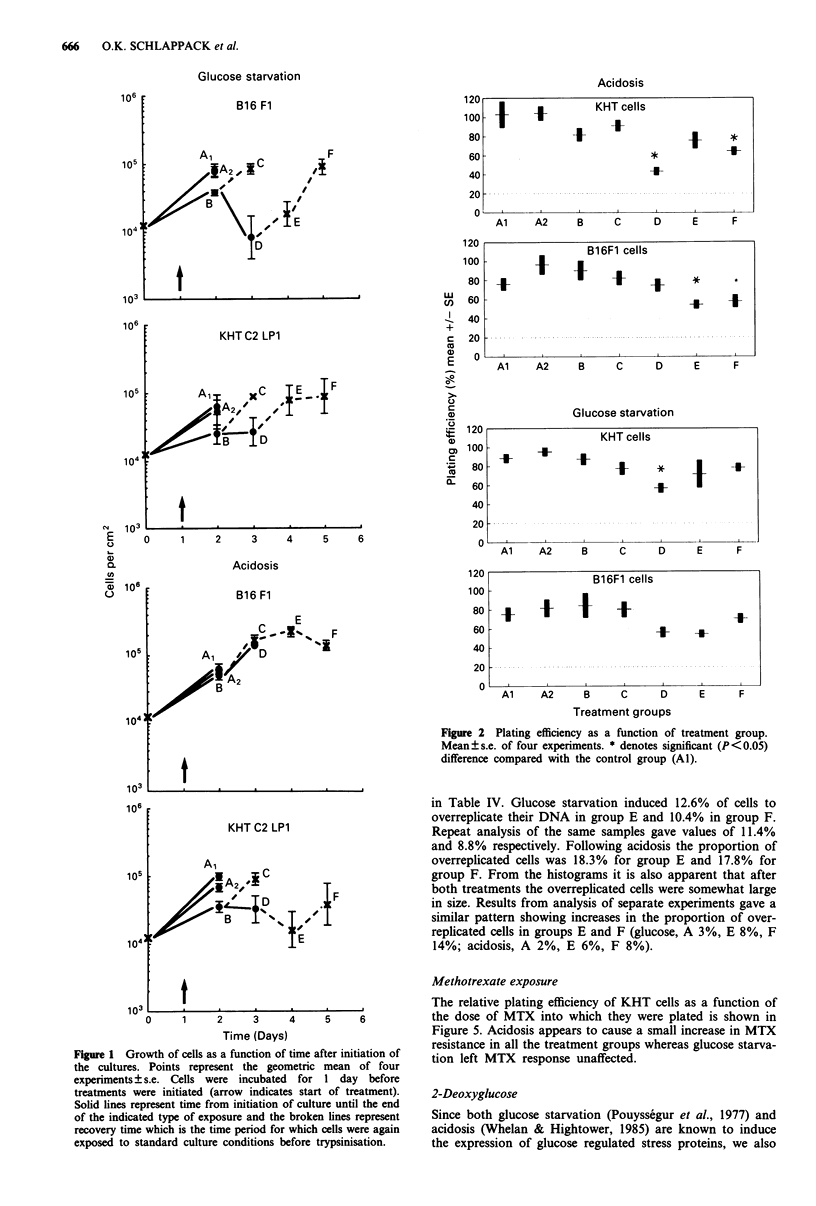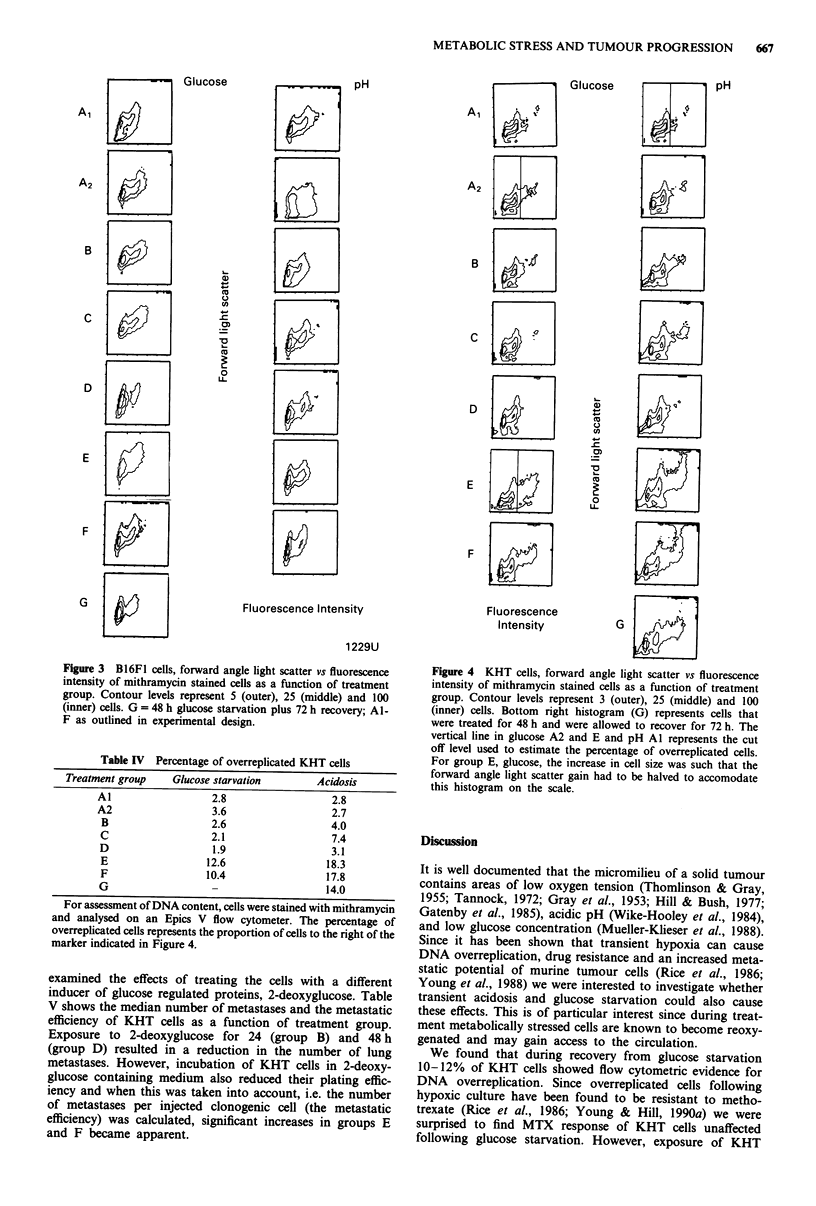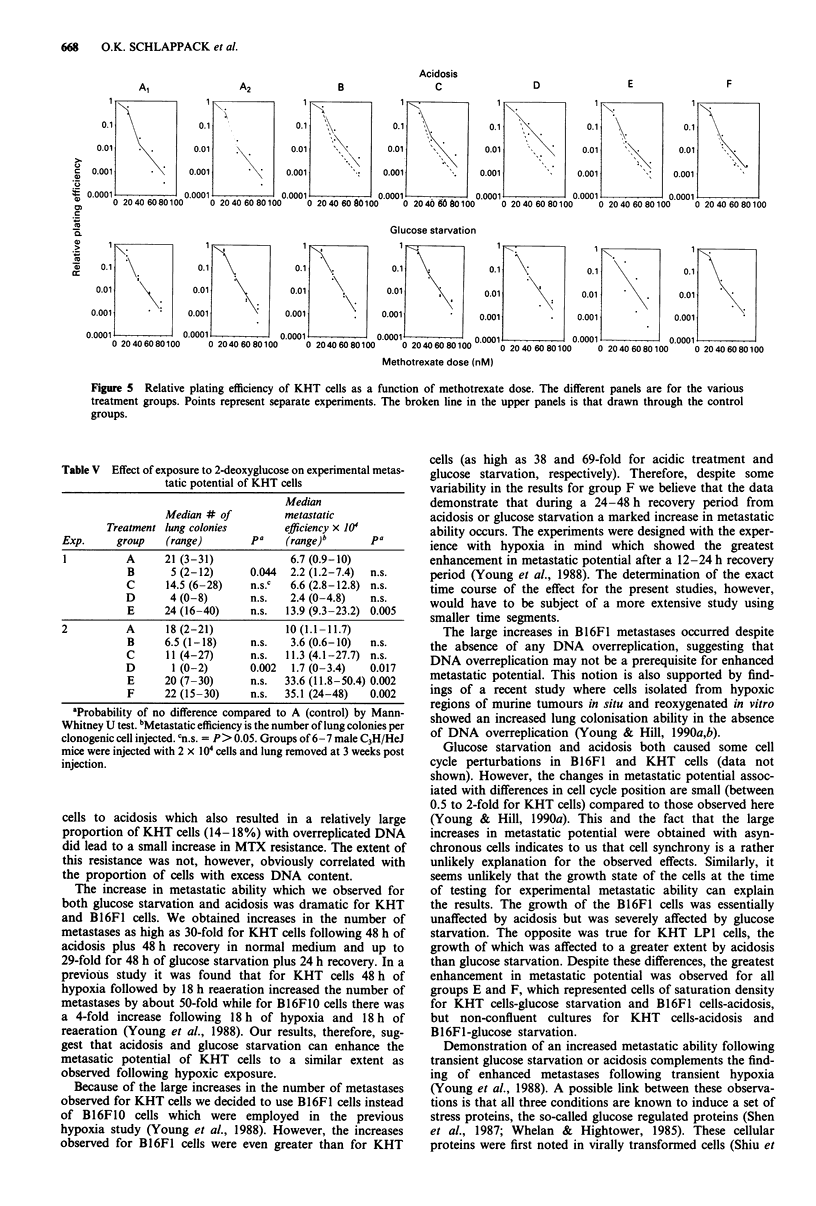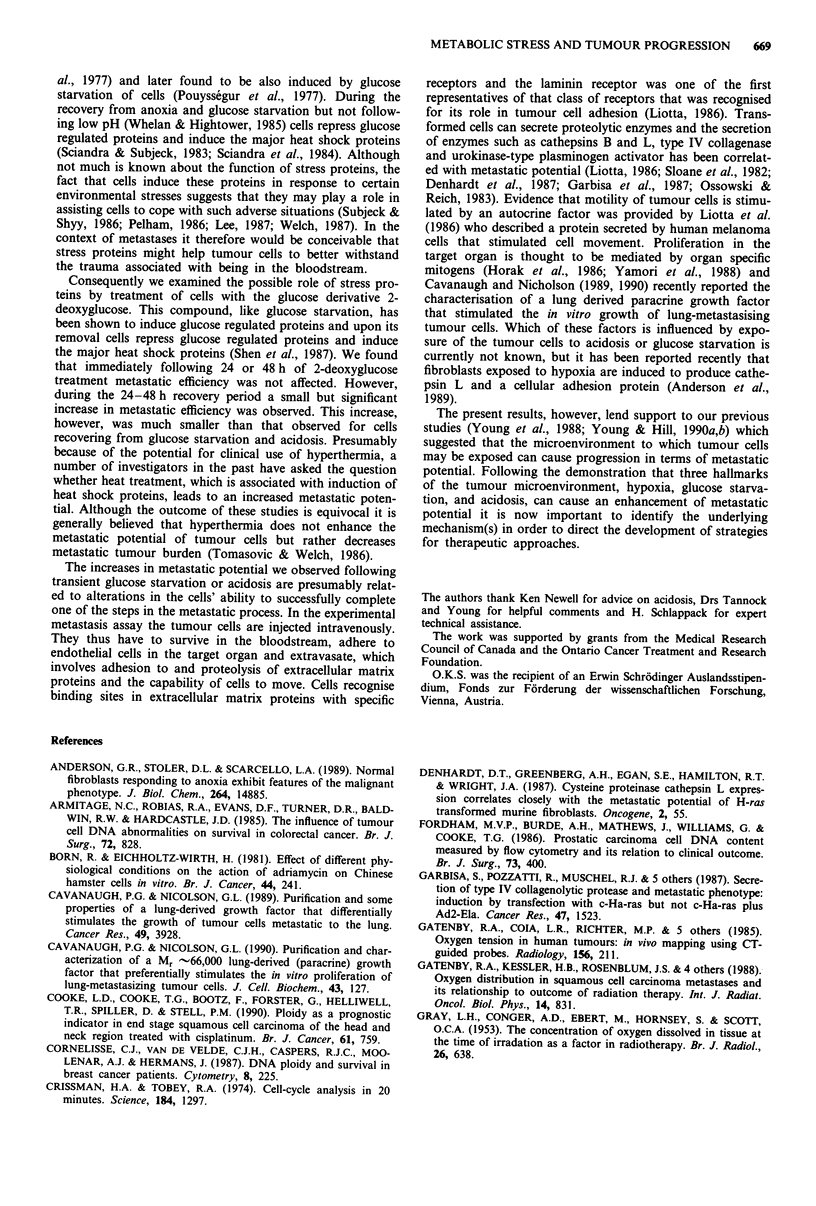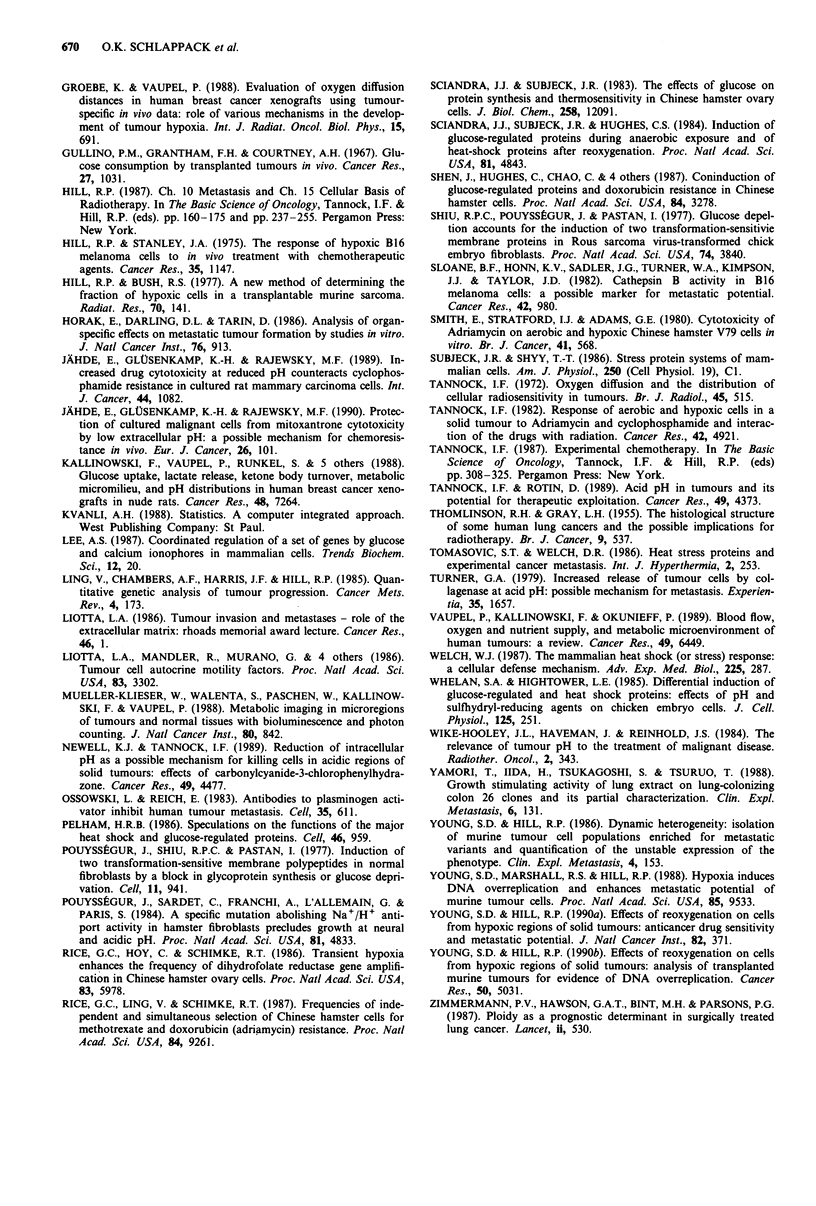# Glucose starvation and acidosis: effect on experimental metastatic potential, DNA content and MTX resistance of murine tumour cells.

**DOI:** 10.1038/bjc.1991.378

**Published:** 1991-10

**Authors:** O. K. Schlappack, A. Zimmermann, R. P. Hill

**Affiliations:** Ontario Cancer Institute, University of Toronto, Canada.

## Abstract

Exposure to oxygen deprivation in vitro has been reported to cause drug resistance in CHO cells (Rice et al., 1986; PNAS 83, 5978) and enhancement of experimental metastatic (colonisation) ability of murine tumour cells (Young et al., 1988; PNAS 85, 9533). Both these studies also demonstrated the induction of a subpopulation of cells with excess DNA content. Since the micromilieu in tumours results in exposure of the tumour cells to conditions of acid pH and nutrient deprivation, as well as hypoxia, we have examined the effect of exposure to acidosis (pH 6.5) and glucose starvation on drug resistance, cellular DNA content and the experimental metastatic ability of KHT sarcoma and B16F1 melanoma cells. Cells were exposed to these conditions for 24 and 48 h and tested for resistance to methotrexate (MTX) or experimental metastatic ability either immediately following these exposures or after 24 or 48 h of recovery in normal growth medium. Both cell lines demonstrated an enhancement of colonisation potential, which was most marked when cells were injected after 48 h of exposure followed by a 24 or 48 h recovery period. Flow cytometric analysis demonstrated an increase in the fraction of KHT cells with excess DNA following both glucose starvation and acidosis we observed only a small increase in MTX resistance following acidic exposure of cells and no change following glucose starvation. Since both acidosis and glucose starvation are known to induce glucose regulated proteins (grp), a subset of the stress protein family, we studied the effect of treatment with another known inducer, 2-deoxyglucose. We found that this agent affected the metastatic efficiency of KHT cells in a manner similar to that observed following exposure to glucose starvation and acidosis. However, further studies are required to establish what role, if any, grp play in this effect. In conclusion this study shows that transient exposure of murine tumour cells to an acidic or glucose deprived environment can cause progression in terms of metastatic potential.


Br. J. Cancer (1991), 64, 663-670 © Macmillan Press Ltd., 1991

### Glucose starvation and acidosis: effect on experimental metastatic potential, DNA content and MTX resistance of murine tumour cells

O.K. Schlappack, A. Zimmermann & R.P. Hill

*The Ontario Cancer Institute and Department of Medical Biophysics, University of Toronto, Toronto, Canada M4X 1K9.*

**Summary** Exposure to oxygen deprivation *in vitro* has been reported to cause drug resistance in CHO cells (Rice *et al.*, 1986; PNAS 83, 5978) and enhancement of experimental metastatic (colonisation) ability of murine tumour cells (Young *et al.*, 1988; PNAS 85, 9533). Both these studies also demonstrated the induction of a subpopulation of cells with excess DNA content. Since the micromilieu in tumours results in exposure of the tumour cells to conditions of acid pH and nutrient deprivation, as well as hypoxia, we have examined the effect of exposure to acidosis (pH 6.5) and glucose starvation on drug resistance, cellular DNA content and the experimental metastatic ability of KHT sarcoma and B16F1 melanoma cells. Cells were exposed to these conditions for 24 and 48 h and tested for resistance to methotrexate (MTX) or experimental metastatic ability either immediately following these exposures or after 24 or 48 h of recovery in normal growth medium. Both cell lines demonstrated an enhancement of colonisation potential, which was most marked when cells were injected after 48 h of exposure followed by a 24 or 48 h recovery period. Flow cytometric analysis demonstrated an increase in the proportion of cells with excess DNA content for KHT cells but not for B16F1 cells. Despite this increase in the fraction of KHT cells with excess DNA following both glucose starvation and acidosis we observed only a small increase in MTX resistance following acidic exposure of cells and no change following glucose starvation. Since both acidosis and glucose starvation are known to induce glucose regulated proteins (grp), a subset of the stress protein family, we studied the effect of treatment with another known inducer, 2-deoxyglucose. We found that this agent affected the metastatic efficiency of KHT cells in a manner similar to that observed following exposure to glucose starvation and acidosis. However, further studies are required to establish what role, if any, grp play in this effect. In conclusion this study shows that transient exposure of murine tumour cells to an acidic or glucose deprived environment can cause progression in terms of metastatic potential.

A malignant tumour is normally characterised by the invasive capacity of its cells. Thus during the local growth of a solid tumour a large number of cells may gain access to blood vessels and a small fraction of these cells will grow at a distant site to form metastases (Hill, 1987). Whereas a small locally growing tumour is potentially curable by surgical removal or radiotherapy, patients with disseminated disease require systemic therapy usually in the form of chemotherapy. Some tumour entities such as testicular, small cell lung, ovary, and mammary cancer are initially responsive to chemotherapy but eventually develop drug resistance (Tannock, 1987). Therefore, the capacity of tumour cells to disseminate in the host and become drug resistant is a major obstacle to therapeutic management. We have been interested in how the tumour microenvironment might influence these cellular properties.

It has been known for some time that in solid tumours regions at a distance from functional vasculature may become necrotic as a result of poor nutrient supply and of poor catabolite removal (Groebe & Vaupel, 1988; Vaupel *et al.*, 1989). Furthermore, there is strong evidence for the presence of hypoxic cells in tumours (Hill, 1987). These cells are less radiosensitive than well oxygenated cells and a recent clinical study (Gatenby *et al.*, 1988) has demonstrated a correlation between measured oxygen levels in tumours and the response of the tumours to radiation therapy. It is also documented that hypoxic cells respond less well to some chemotherapeutic agents, both *in vitro* (Smith *et al.*, 1980; Born & Eichholtz-Wirth, 1981) and *in vivo* (Hill & Stanley, 1975; Tannock, 1982).

Recently, Rice *et al.* (1986) reported increased MTX resistance of Chinese hamster ovary cells following transient hypoxic exposure. They also found that this transient oxygen deprivation induced a subpopulation of cells with overreplicated DNA (i.e. cells with more than $G_2/M$ DNA content), and that these cells were the most drug resistant. Following this observation Young *et al.* (1988) and Young & Hill (1990*a*) investigated the consequences of transient oxygen deprivation in terms of the metastatic potential of murine tumour cells. Using three murine tumour cell lines (KHT, SCCVII and B16F10) they found that transient hypoxia enhanced the metastatic potential and that this was associated with the generation of a subpopulation of overreplicated cells. Although this association is not specific, it is consistent with the finding that abnormal cellular DNA content can be associated with a reduced life expectancy in a number of human cancers (Cornelisse *et al.*, 1987; Zimmermann *et al.*, 1987; Armitage *et al.*, 1985; Fordham *et al.*, 1986; Cooke *et al.*, 1990). It can be argued that overreplicated cells may give rise to cells with amplified genes that code for metastasis–relevant products (Ling *et al.*, 1985) since it has been shown that cells resistant to MTX or doxorubicin due to amplification to the genes coding for DHFR or p-glycoprotein respectively were derived from overreplicated cells (Rice *et al.*, 1987).

The micromilieu of a solid tumour is not only characterised by hypoxia. As a result of lactic acid production and hydrolysis of ATP the pH in tumours is found to be acidic (range: 5.8–7.5 in rodents and 5.85–7.68 in humans) and generally lower than in surrounding normal tissue (mean subcutis value for man, rats, and dogs is: 7.5, 7.4 and 7.3 respectively, Tannock & Rotin, 1989; Wike-Hooley *et al.*, 1984). Studies on the influence of pH on the radiation response of cells show a slightly decreased radiation sensitivity towards lower extracellular pH (Wike-Hooley *et al.*, 1984). The cytotoxic action of doxorubicin (Born & Eichholtz-Wirth, 1981) and mitoxantrone (Jähde *et al.*, 1990) is also reduced at low extracellular pH whereas the cytotoxicity of cyclophosphamide increases at reduced pH (Jähde *et al.*, 1989). The observation that the ability of collagenase to disaggregate a solid metastasising lymphosarcoma increases with reduced environmental pH has been used to implicate low pH in metastasis (Turner, 1979).

*In vivo* neoplastic tissues utilise a large quantity of glucose. This is evidenced by the observation that the normal sub-

Correspondence: R.P. Hill, The Ontario Cancer Institute, 500 Sherbourne Street, Toronto, Ontario, Canada M4X 1K9.
Received 25 March 1991; and in revised form 4 June 1991.

cutaneous interstitial fluid of rats contains 90–110 mg glucose per 100 ml (plasma levels: 135–160 mg $ml^{-1}$) whereas the interstitial fluid of Walker carcinomas was found to contain only between 0 and 5 mg glucose per ml (Gullino *et al.*, 1967). Thus, apparently all glucose passing through the tumour vascular wall is rapidly utilised by neoplastic cells. Recently, Kallinowski *et al.* (1988) reported extended areas of very low glucose concentrations in a human breast cancer xenograft as assessed by metabolic imaging with bioluminescence and photon counting (Mueller-Klieser *et al.*, 1988).

In this project we were interested to investigate whether these other stress conditions (acidosis and glucose starvation), to which tumour cells may be exposed, have an influence on tumour progression. Using two murine tumour cell lines (KHT and B16F1) we found that transient glucose starvation and acidosis can cause a marked enhancement in experimental metastases and overreplication of cellular DNA.

### Materials and methods

### *Cells*

KHT C2 LP1 and B16F1 cells were routinely cultivated in alpha MEM medium (α-MEM, Gibco, Grand Island, NY, USA) containing 0.1 g $l^{-1}$ penicillin and 0.1 g $l^{-1}$ streptomycin and supplemented with 10% foetal bovine serum (FBS, Gibco). They were incubated in humidified air containing 5% $CO_2$ at 37°C. The origin of the KHT C2 LP1 cell line has been described previously (Young & Hill, 1986) and the B16F1 cells have been kindly provided (in 1980) by Dr Fidler (Department of Cell Biology, University of Texas, M.D. Anderson Cancer Center).

### *Glucose starvation and acidosis*

*Glucose starvation* Cells were exposed to alpha MEM medium containing no glucose. Before use the medium was supplemented with 10% dialysed foetal bovine serum.

*Acidosis* Alpha MEM medium containing either 25 mM $NaHCO_3$ or 25 mM Hepes was prepared and the desired pH of 6.5 obtained by mixing Hepes and $NaHCO_3$ containing medium as described by Newell and Tannock (1989). Medium was supplemented with 10% FBS and 0.1 mM hypoxanthine and 0.1 mM uridine as described by Pouysségur *et al.* (1984) for $HCO_3$-free medium. The pH, measured using a PHM 82 Standard pH meter (Radiometer, Copenhagen, Denmark) following calibration with certified reference buffer solutions (Fisher Scientific, Farr Lawn, New Jersey, USA), was found to remain essentially constant during cell exposure, i.e. a 48 h incubation of cells only resulted in an increase in pH between 0.1–0.2 pH units.

*2-Deoxyglucose* Cells were exposed to alpha MEM medium (supplemented with 10% FBS) containing 10 mM 2-deoxyglucose (Sigma Chemical Company, St. Louis, MO).

### *Experimental design*

Table I shows the experimental design used for all three treatments (acidosis, glucose starvation and 2-deoxyglucose). This design was chosen based on the experience with transient exposure of cells to hypoxia which showed maximal enhancement of metastatic potential following 24 and 48 h of hypoxia plus 18 h recovery (Young *et al.*, 1988).

Cells were plated into 80 $mm^2$ tissue culture flasks at $10^6$ cells per flask and allowed 24 h under normal growth conditions before the start of the treatment. Following the stated exposure times, the cells were trypsinised, counted with the aid of a Neubauer hemocytometer and appropriately diluted for i.v. injections, plating in MTX or storage for flow cytometry. Cells of control group A1 were always exposed to regular alpha MEM medium supplemented with 10% FBS whereas A2 control cells were exposed to alpha MEM medium plus 10% dialysed foetal bovine serum in the case of glucose starvation and $NaHCO_3$ repleted alpha MEM medium plus 10% FBS in the case of acidic treatment. The control cells were exposed for 24 h before testing.

**Table I** Experimental design

| | |
|---|---|
| A1 | control |
| A2 | control |
| B | 24 h treatment, no recovery |
| C | 24 h treatment, 24 h recovery |
| D | 48 h treatment, no recovery |
| E | 48 h treatment, 24 h recovery |
| F | 48 h treatment, 48 h recovery |

### *Methotrexate incubation*

Commercial methotrexate (Cyanamid GmbH, Wolfratshausen, Germany) was diluted with PBS to 0.025 mg $ml^{-1}$ and final dilutions were made in alpha MEM medium devoid of nucleosides but supplemented with 10% dialysed foetal bovine serum. Cells were plated in 100 mm tissue culture dishes containing 0, 20, 40, 60 or 80 nM MTX. At 10–14 days the cells were fixed and stained with methylene blue, the number of colonies was counted and the relative plating efficiency was calculated.

### *Flow cytometry*

*Storage and staining of cells* For assessment of DNA content the cells were fixed and stored in 70% methanol at 4°C for 2–4 weeks before being stained with mithramycin. Mithramycin (100 μg $ml^{-1}$) was dissolved in 25% ethanol containing 15 mM $MgCl_2$. Immediately prior to analysis, the cells were centrifuged, the supernatant methanol discarded and cells resuspended in mithramycin solution as described by Crissman and Tobey (1974).

*Analysis* Analysis was carried out on an Epics V flow cytometer (Coulter Electronics, Hialeah, FL) operating at a laser power of 150 mW and at an excitation wavelength of 457 nm. Green fluorescence (a measure of DNA content), collected with a 525 band-pass filter, and forward angle light scatter (an estimate of cell size) were acquired in list mode for 20,000 cells per sample.

### *Animals*

Unless otherwise stated female syngeneic C3H/CRL mice were used for KHT C2 LP1 cells and C57Bl/CRL mice for B16F1 cells. Mice were supplied by Charles River Canada Inc. (St Constant, Quebec) and kept in the animal colony of this Institution at a 12 h light and 12 h dark cycle. Mice were allowed food and water *ad libitum* and were used in experiments at a mean body weight of 20 g.

### *Metastases*

The experimental metastatic ability of the cells was tested by injecting $5 \times 10^4$ cells (unless otherwise stated) into the lateral tail vein of groups of 6–7 mice. At 21 or 23 days after tumour cell injection the animals were killed by cervical dislocation or $CO_2$ asphyxiation, the lungs were removed and fixed in Bouin's solution. The number of surface lung metastases was counted with the aid of a dissecting microscope as previously described (Young & Hill, 1986; 1990*a*,*b*; Young *et al.*, 1988).

### *Data analysis*

The data was analysed using Minitab (Minitab Inc., State College, PA) and SAS statistics (SAS Institute Inc, Cary, NC). The groups of an experiment were first compared with the Kruskal-Wallis test and when a significant difference with alpha $\leqslant 0.05$ was found, the treatment groups were compared with the control group using the Mann-Whitney U test (Kvanli, 1988).

### Results

### *Metastases*

The effect of exposure to acidosis and to glucose starvation on the experimental metastatic potential of KHT and B16F1 cells is shown in Tables II and III, respectively. Acidosis caused an increase in the number of KHT lung metastases when cells were allowed to recover from acidosis before injection. The effect was greatest for treatment groups E and F (i.e. 24 and 48 h following 48 h of acidic exposure). For B16F1 cells, the results were similar with marked increases in groups E and F. Glucose starvation resulted in an enhancement of KHT metastatic ability following all exposure schedules. However, the effect was greatest for groups D-F. B16F1 cells also demonstrated an increase in metastasis formation in all treatment groups. The effect was greatest for group E, i.e. 24 h after a 48 h glucose starvation period. There is some variability in the number of metastases observed in the two control groups (A1 and A2), particularly for the experiments involving exposure to acidosis. These differences were not consistent but, because of them, significance testing was done *vs* the two control groups independently when a significant difference between A1 and A2 was observed. These differences in the control groups do not affect the conclusion from these experiments that an increase in metastasis formation is seen, particularly in groups E and F.

### *Influence of glucose starvation and acidosis on cell growth and plating efficiency*

*Cell growth* Figure 1 shows the number of cells recovered from the growth flasks as a function of time after initiation of the cultures for the two cell lines and the two treatments. Cells were seeded at a density of $1.25 \times 10^4$ per $cm^2$ ($10^6$ cells/flask) in T80 tissue culture flasks on day 0 and on day 1, treatment was started by replacing alpha MEM medium with the indicated treatment medium. Glucose starvation impaired the growth of KHT cells but to a lesser extent than that of B16F1 cells. Acidosis left B16F1 cell growth uninhibited whereas it markedly affected KHT cell growth.

**Table II** Effect of exposure to acidosis on experimental metastatic potential of KHT and B16F1 cells

| *Cell line* | *Exp.* | *Treatment group* | *Median no. of lung metastases* | $P^a$ | $P^b$ |
|---|---|---|---|---|---|
| KHT | | A1 | 3 (0–12)[c] | | |
| | 1 | A2 | 0 (0–14) | n.s. | |
| | | B | 7 (1–35) | 0.034 | |
| | | C | 15 (1–23) | 0.046 | |
| | | D | 1 (0–7) | n.s. | |
| | | E | 60 (22–>100) | 0.003 | |
| | | F | 13 (0–27) | n.s. | |
| | 2 | A1 | 3 (0–5) | | 0.008 |
| | | A2 | 12 (2–27) | 0.008 | |
| | | B | 8 (1–35) | n.s. | n.s. |
| | | C | 26 (3–33) | 0.016 | n.s. |
| | | D | 3 (0–11) | n.s. | 0.038 |
| | | E | 32 (6–60) | 0.002 | n.s. |
| | | F | 91.5 (12–122) | 0.002 | 0.012 |
| B16F1 | 1 | A1 | 20 (10–32) | | 0.002 |
| | | A2 | 1 (0–3) | 0.002 | |
| | | B | 1 (1–5) | 0.002 | n.s. |
| | | C | 8 (5–27) | n.s. | 0.002 |
| | | D | 5 (2–13) | 0.004 | 0.004 |
| | | E | 87 (60–107) | 0.002 | 0.002 |
| | 2 | A1 | 3 (1–14) | | |
| | | A2 | 15 (0–35) | n.s. | |
| | | B | 7 (3–32) | n.s. | |
| | | C | 30 (11–39) | 0.003 | |
| | | D | 27 (11–38) | 0.006 | |
| | | E | 87.5 (56–96) | 0.003 | |
| | | F | 115 (98–145) | 0.002 | |

[a]Probability of no difference compared to A1 control by Mann-Whitney U test. [b]Probability of no difference compared to A2 control by Mann-Whitney U test. [c]Numbers in parentheses, range.

**Table III** Effect of exposure to glucose starvation on experimental metastatic potential of KHT and B16F1 cells

| *Cell line* | *Exp.* | *Treatment group* | *Median no. of lung metastases* | $P^a$ | $P^b$ |
|---|---|---|---|---|---|
| KHT | 1 | A1 | 3 (1–6)[c] | | |
| | | A2 | 3 (0–5) | n.s. | |
| | | B | 21 (11–53) | 0.002 | |
| | | C | 16 (3–21) | 0.007 | |
| | | D | 55 (0–100) | 0.025 | |
| | | E | 86 (35–100) | 0.002 | |
| | 2 | A1 | 13 (0–23) | | |
| | | A2 | 13 (0–19) | n.s. | |
| | | B | 24 (4–58) | n.s. | |
| | | C | 61 (18–115) | 0.007 | |
| | | D | 62 (11–115) | 0.015 | |
| | | E | 156 (2–165) | 0.022 | |
| | | F | 97 (0–120) | 0.031 | |
| B16F1 | 1 | A1 | 0 (0–3) | | |
| | | A2 | 1.5 (0–73) | n.s. | |
| | | B | 23.5 (12–36) | 0.003 | |
| | | C | 19 (12–39) | 0.002 | |
| | | D | 23 (18–47) | 0.002 | |
| | | E | 104 (57–118) | 0.002 | |
| | | F | 3 (1–11) | 0.013 | |
| | 2 | A1 | 5 (3–9) | | 0.018 |
| | | A2 | 2 (0–5) | 0.018 | |
| | | B | 14 (2–22) | n.s. | 0.007 |
| | | C | 70 (40–100) | 0.002 | 0.002 |
| | | D | 39 (15–58) | 0.002 | 0.002 |
| | | E | 50 (23–65) | 0.002 | 0.002 |
| | | F | 23 (11–34) | 0.002 | 0.002 |

[a]Probability of no difference compared to A1 control by Mann-Whitney U test. [b]Probability of no difference compared to A2 control by Mann-Whitney U test. [c]Numbers of parentheses, range.

*Plating efficiency (PE)* Figure 2 shows PE for cells from the various treatment groups for both cell lines. Acidosis caused a significant reduction in PE in group D and F of KHT cells and in groups E for B16F1 cells. Glucose starvation only significantly reduced plating efficiency in group D of KHT cells. Therefore, for these groups the increase in experimental metastatic efficiency, EME (i.e. the number of lung colonies per clonogenic cell injected) for the various treatment groups is greater than the observed increases in number of lung metastases.

### *Flow cytometry*

Dual parameter contour plots of forward angle light scatter as a function of fluorescence intensity for B16F1 cells are shown in Figure 3. Following glucose starvation groups C, E and F showed small increases in cell size as estimated by forward angle light scatter. However, there is no indication of a fraction of cells with increased DNA content. In contrast, the same plot for KHT cells, as shown in Figure 4, exhibited a marked increase in the number of cells with more than $G_2/M$ DNA content in groups E and F for glucose starvation and in groups C, E, F and G following acidic exposure.

To estimate the proportion of cells with overreplicated DNA we placed a marker in the histogram of the control group A1 such that 2–3% of cells were to the right of the marker. This percentage is the background proportion of cells with excess DNA content for this cell line as has been reported previously (Young & Hill, 1990*b*). Using the same marker position we then estimated the percentage of overreplicated cells for all treatment groups. The results are given

**Figure 1** Growth of cells as a function of time after initiation of the cultures. Points represent the geometric mean of four experiments ± s.e. Cells were incubated for 1 day before treatments were initiated (arrow indicates start of treatment). Solid lines represent time from initiation of culture until the end of the indicated type of exposure and the broken lines represent recovery time which is the time period for which cells were again exposed to standard culture conditions before trypsinisation.

**Figure 2** Plating efficiency as a function of treatment group. Mean ± s.e. of four experiments. * denotes significant ($P < 0.05$) difference compared with the control group (A1).

in Table IV. Glucose starvation induced 12.6% of cells to overreplicate their DNA in group E and 10.4% in group F. Repeat analysis of the same samples gave values of 11.4% and 8.8% respectively. Following acidosis the proportion of overreplicated cells was 18.3% for group E and 17.8% for group F. From the histograms it is also apparent that after both treatments the overreplicated cells were somewhat large in size. Results from analysis of separate experiments gave a similar pattern showing increases in the proportion of overreplicated cells in groups E and F (glucose, A 3%, E 8%, F 14%; acidosis, A 2%, E 6%, F 8%).

*Methotrexate exposure*

The relative plating efficiency of KHT cells as a function of the dose of MTX into which they were plated is shown in Figure 5. Acidosis appears to cause a small increase in MTX resistance in all the treatment groups whereas glucose starvation left MTX response unaffected.

*2-Deoxyglucose*

Since both glucose starvation (Pouysségur *et al.*, 1977) and acidosis (Whelan & Hightower, 1985) are known to induce the expression of glucose regulated stress proteins, we also

**Figure 3** B16F1 cells, forward angle light scatter *vs* fluorescence intensity of mithramycin stained cells as a function of treatment group. Contour levels represent 5 (outer), 25 (middle) and 100 (inner) cells. G = 48 h glucose starvation plus 72 h recovery; A1–F as outlined in experimental design.

**Table IV** Percentage of overreplicated KHT cells

| *Treatment group* | *Glucose starvation* | *Acidosis* |
|---|---|---|
| A1 | 2.8 | 2.8 |
| A2 | 3.6 | 2.7 |
| B | 2.6 | 4.0 |
| C | 2.1 | 7.4 |
| D | 1.9 | 3.1 |
| E | 12.6 | 18.3 |
| F | 10.4 | 17.8 |
| G | – | 14.0 |

For assessment of DNA content, cells were stained with mithramycin and analysed on an Epics V flow cytometer. The percentage of overreplicated cells represents the proportion of cells to the right of the marker indicated in Figure 4.

examined the effects of treating the cells with a different inducer of glucose regulated proteins, 2-deoxyglucose. Table V shows the median number of metastases and the metastatic efficiency of KHT cells as a function of treatment group. Exposure to 2-deoxyglucose for 24 (group B) and 48 h (group D) resulted in a reduction in the number of lung metastases. However, incubation of KHT cells in 2-deoxyglucose containing medium also reduced their plating efficiency and when this was taken into account, i.e. the number of metastases per injected clonogenic cell (the metastatic efficiency) was calculated, significant increases in groups E and F became apparent.

**Figure 4** KHT cells, forward angle light scatter *vs* fluorescence intensity of mithramycin stained cells as a function of treatment group. Contour levels represent 3 (outer), 25 (middle) and 100 (inner) cells. Bottom right histogram (G) represents cells that were treated for 48 h and were allowed to recover for 72 h. The vertical line in glucose A2 and E and pH A1 represents the cut off level used to estimate the percentage of overreplicated cells. For group E, glucose, the increase in cell size was such that the forward angle light scatter gain had to be halved to accomodate this histogram on the scale.

### Discussion

It is well documented that the micromilieu of a solid tumour contains areas of low oxygen tension (Thomlinson & Gray, 1955; Tannock, 1972; Gray *et al.*, 1953; Hill & Bush, 1977; Gatenby *et al.*, 1985), acidic pH (Wike-Hooley *et al.*, 1984), and low glucose concentration (Mueller-Klieser *et al.*, 1988). Since it has been shown that transient hypoxia can cause DNA overreplication, drug resistance and an increased metastatic potential of murine tumour cells (Rice *et al.*, 1986; Young *et al.*, 1988) we were interested to investigate whether transient acidosis and glucose starvation could also cause these effects. This is of particular interest since during treatment metabolically stressed cells are known to become reoxygenated and may gain access to the circulation.

We found that during recovery from glucose starvation 10–12% of KHT cells showed flow cytometric evidence for DNA overreplication. Since overreplicated cells following hypoxic culture have been found to be resistant to methotrexate (Rice *et al.*, 1986; Young & Hill, 1990*a*) we were surprised to find MTX response of KHT cells unaffected following glucose starvation. However, exposure of KHT

**Figure 5** Relative plating efficiency of KHT cells as a function of methotrexate dose. The different panels are for the various treatment groups. Points represent separate experiments. The broken line in the upper panels is that drawn through the control groups.

**Table V** Effect of exposure to 2-deoxyglucose on experimental metastatic potential of KHT cells

| *Exp.* | *Treatment group* | *Median # of lung colonies (range)* | $P^a$ | *Median metastatic efficiency* × $10^4$ *(range)*$^b$ | $P^a$ |
|---|---|---|---|---|---|
| 1 | A | 21 (3–31) | | 6.7 (0.9–10) | |
| | B | 5 (2–12) | 0.044 | 2.2 (1.2–7.4) | n.s. |
| | C | 14.5 (6–28) | n.s.$^c$ | 6.6 (2.8–12.8) | n.s. |
| | D | 4 (0–8) | n.s. | 2.4 (0–4.8) | n.s. |
| | E | 24 (16–40) | n.s. | 13.9 (9.3–23.2) | 0.005 |
| 2 | A | 18 (2–21) | | 10 (1.1–11.7) | |
| | B | 6.5 (1–18) | n.s. | 3.6 (0.6–10) | n.s. |
| | C | 11 (4–27) | n.s. | 11.3 (4.1–27.7) | n.s. |
| | D | 1 (0–2) | 0.002 | 1.7 (0–3.4) | 0.017 |
| | E | 20 (7–30) | n.s. | 33.6 (11.8–50.4) | 0.002 |
| | F | 22 (15–30) | n.s. | 35.1 (24–48) | 0.002 |

[a]Probability of no difference compared to A (control) by Mann-Whitney U test. [b]Metastatic efficiency is the number of lung colonies per clonogenic cell injected. [c]n.s. = $P > 0.05$. Groups of 6–7 male $C_3H$/HeJ mice were injected with $2 \times 10^4$ cells and lung removed at 3 weeks post injection.

cells to acidosis which also resulted in a relatively large proportion of KHT cells (14–18%) with overreplicated DNA did lead to a small increase in MTX resistance. The extent of this resistance was not, however, obviously correlated with the proportion of cells with excess DNA content.

The increase in metastatic ability which we observed for both glucose starvation and acidosis was dramatic for KHT and B16F1 cells. We obtained increases in the number of metastases as high as 30-fold for KHT cells following 48 h of acidosis plus 48 h recovery in normal medium and up to 29-fold for 48 h of glucose starvation plus 24 h recovery. In a previous study it was found that for KHT cells 48 h of hypoxia followed by 18 h reaeration increased the number of metastases by about 50-fold while for B16F10 cells there was a 4-fold increase following 18 h of hypoxia and 18 h of reaeration (Young *et al.*, 1988). Our results, therefore, suggest that acidosis and glucose starvation can enhance the metasatic potential of KHT cells to a similar extent as observed following hypoxic exposure.

Because of the large increases in the number of metastases observed for KHT cells we decided to use B16F1 cells instead of B16F10 cells which were employed in the previous hypoxia study (Young *et al.*, 1988). However, the increases observed for B16F1 cells were even greater than for KHT cells (as high as 38 and 69-fold for acidic treatment and glucose starvation, respectively). Therefore, despite some variability in the results for group F we believe that the data demonstrate that during a 24–48 h recovery period from acidosis or glucose starvation a marked increase in metastatic ability occurs. The experiments were designed with the experience with hypoxia in mind which showed the greatest enhancement in metastatic potential after a 12–24 h recovery period (Young *et al.*, 1988). The determination of the exact time course of the effect for the present studies, however, would have to be subject of a more extensive study using smaller time segments.

The large increases in B16F1 metastases occurred despite the absence of any DNA overreplication, suggesting that DNA overreplication may not be a prerequisite for enhanced metastatic potential. This notion is also supported by findings of a recent study where cells isolated from hypoxic regions of murine tumours *in situ* and reoxygenated *in vitro* showed an increased lung colonisation ability in the absence of DNA overreplication (Young & Hill, 1990*a*,*b*).

Glucose starvation and acidosis both caused some cell cycle perturbations in B16F1 and KHT cells (data not shown). However, the changes in metastatic potential associated with differences in cell cycle position are small (between 0.5 to 2-fold for KHT cells) compared to those observed here (Young & Hill, 1990*a*). This and the fact that the large increases in metastatic potential were obtained with asynchronous cells indicates to us that cell synchrony is a rather unlikely explanation for the observed effects. Similarly, it seems unlikely that the growth state of the cells at the time of testing for experimental metastatic ability can explain the results. The growth of the B16F1 cells was essentially unaffected by acidosis but was severely affected by glucose starvation. The opposite was true for KHT LP1 cells, the growth of which was affected to a greater extent by acidosis than glucose starvation. Despite these differences, the greatest enhancement in metastatic potential was observed for all groups E and F, which represented cells of saturation density for KHT cells-glucose starvation and B16F1 cells-acidosis, but non-confluent cultures for KHT cells-acidosis and B16F1-glucose starvation.

Demonstration of an increased metastatic ability following transient glucose starvation or acidosis complements the finding of enhanced metastases following transient hypoxia (Young *et al.*, 1988). A possible link between these observations is that all three conditions are known to induce a set of stress proteins, the so-called glucose regulated proteins (Shen *et al.*, 1987; Whelan & Hightower, 1985). These cellular proteins were first noted in virally transformed cells (Shiu *et*

al., 1977) and later found to be also induced by glucose starvation of cells (Pouysségur et al., 1977). During the recovery from anoxia and glucose starvation but not following low pH (Whelan & Hightower, 1985) cells repress glucose regulated proteins and induce the major heat shock proteins (Sciandra & Subjeck, 1983; Sciandra et al., 1984). Although not much is known about the function of stress proteins, the fact that cells induce these proteins in response to certain environmental stresses suggests that they may play a role in assisting cells to cope with such adverse situations (Subjeck & Shyy, 1986; Pelham, 1986; Lee, 1987; Welch, 1987). In the context of metastases it therefore would be conceivable that stress proteins might help tumour cells to better withstand the trauma associated with being in the bloodstream.

Consequently we examined the possible role of stress proteins by treatment of cells with the glucose derivative 2-deoxyglucose. This compound, like glucose starvation, has been shown to induce glucose regulated proteins and upon its removal cells repress glucose regulated proteins and induce the major heat shock proteins (Shen et al., 1987). We found that immediately following 24 or 48 h of 2-deoxyglucose treatment metastatic efficiency was not affected. However, during the 24–48 h recovery period a small but significant increase in metastatic efficiency was observed. This increase, however, was much smaller than that observed for cells recovering from glucose starvation and acidosis. Presumably because of the potential for clinical use of hyperthermia, a number of investigators in the past have asked the question whether heat treatment, which is associated with induction of heat shock proteins, leads to an increased metastatic potential. Although the outcome of these studies is equivocal it is generally believed that hyperthermia does not enhance the metastatic potential of tumour cells but rather decreases metastatic tumour burden (Tomasovic & Welch, 1986).

The increases in metastatic potential we observed following transient glucose starvation or acidosis are presumably related to alterations in the cells' ability to successfully complete one of the steps in the metastatic process. In the experimental metastasis assay the tumour cells are injected intravenously. They thus have to survive in the bloodstream, adhere to endothelial cells in the target organ and extravasate, which involves adhesion to and proteolysis of extracellular matrix proteins and the capability of cells to move. Cells recognise binding sites in extracellular matrix proteins with specific receptors and the laminin receptor was one of the first representatives of that class of receptors that was recognised for its role in tumour cell adhesion (Liotta, 1986). Transformed cells can secrete proteolytic enzymes and the secretion of enzymes such as cathepsins B and L, type IV collagenase and urokinase-type plasminogen activator has been correlated with metastatic potential (Liotta, 1986; Sloane et al., 1982; Denhardt et al., 1987; Garbisa et al., 1987; Ossowski & Reich, 1983). Evidence that motility of tumour cells is stimulated by an autocrine factor was provided by Liotta et al. (1986) who described a protein secreted by human melanoma cells that stimulated cell movement. Proliferation in the target organ is thought to be mediated by organ specific mitogens (Horak et al., 1986; Yamori et al., 1988) and Cavanaugh and Nicholson (1989, 1990) recently reported the characterisation of a lung derived paracrine growth factor that stimulated the in vitro growth of lung-metastasising tumour cells. Which of these factors is influenced by exposure of the tumour cells to acidosis or glucose starvation is currently not known, but it has been reported recently that fibroblasts exposed to hypoxia are induced to produce cathepsin L and a cellular adhesion protein (Anderson et al., 1989).

The present results, however, lend support to our previous studies (Young et al., 1988; Young & Hill, 1990a,b) which suggested that the microenvironment to which tumour cells may be exposed can cause progression in terms of metastatic potential. Following the demonstration that three hallmarks of the tumour microenvironment, hypoxia, glucose starvation, and acidosis, can cause an enhancement of metastatic potential it is now important to identify the underlying mechanism(s) in order to direct the development of strategies for therapeutic approaches.

The authors thank Ken Newell for advice on acidosis, Drs Tannock and Young for helpful comments and H. Schlappack for expert technical assistance.

The work was supported by grants from the Medical Research Council of Canada and the Ontario Cancer Treatment and Research Foundation.

O.K.S. was the recipient of an Erwin Schrödinger Auslandsstipendium, Fonds zur Förderung der wissenschaftlichen Forschung, Vienna, Austria.

### References

ANDERSON, G.R., STOLER, D.L. & SCARCELLO, L.A. (1989). Normal fibroblasts responding to anoxia exhibit features of the malignant phenotype. *J. Biol. Chem.*, **264**, 14885.

ARMITAGE, N.C., ROBIAS, R.A., EVANS, D.F., TURNER, D.R., BALDWIN, R.W. & HARDCASTLE, J.D. (1985). The influence of tumour cell DNA abnormalities on survival in colorectal cancer. *Br. J. Surg.*, **72**, 828.

BORN, R. & EICHHOLTZ-WIRTH, H. (1981). Effect of different physiological conditions on the action of adriamycin on Chinese hamster cells *in vitro*. *Br. J. Cancer*, **44**, 241.

CAVANAUGH, P.G. & NICOLSON, G.L. (1989). Purification and some properties of a lung-derived growth factor that differentially stimulates the growth of tumour cells metastatic to the lung. *Cancer Res.*, **49**, 3928.

CAVANAUGH, P.G. & NICOLSON, G.L. (1990). Purification and characterization of a $M_r \sim 66{,}000$ lung-derived (paracrine) growth factor that preferentially stimulates the *in vitro* proliferation of lung-metastasizing tumour cells. *J. Cell. Biochem.*, **43**, 127.

COOKE, L.D., COOKE, T.G., BOOTZ, F., FORSTER, G., HELLIWELL, T.R., SPILLER, D. & STELL, P.M. (1990). Ploidy as a prognostic indicator in end stage squamous cell carcinoma of the head and neck region treated with cisplatinum. *Br. J. Cancer*, **61**, 759.

CORNELISSE, C.J., VAN DE VELDE, C.J.H., CASPERS, R.J.C., MOOLENAR, A.J. & HERMANS, J. (1987). DNA ploidy and survival in breast cancer patients. *Cytometry*, **8**, 225.

CRISSMAN, H.A. & TOBEY, R.A. (1974). Cell-cycle analysis in 20 minutes. *Science*, **184**, 1297.

DENHARDT, D.T., GREENBERG, A.H., EGAN, S.E., HAMILTON, R.T. & WRIGHT, J.A. (1987). Cysteine proteinase cathepsin L expression correlates closely with the metastatic potential of H-*ras* transformed murine fibroblasts. *Oncogene*, **2**, 55.

FORDHAM, M.V.P., BURDE, A.H., MATHEWS, J., WILLIAMS, G. & COOKE, T.G. (1986). Prostatic carcinoma cell DNA content measured by flow cytometry and its relation to clinical outcome. *Br. J. Surg.*, **73**, 400.

GARBISA, S., POZZATTI, R., MUSCHEL, R.J. & 5 others (1987). Secretion of type IV collagenolytic protease and metastatic phenotype: induction by transfection with c-Ha-ras but not c-Ha-ras plus Ad2-Ela. *Cancer Res.*, **47**, 1523.

GATENBY, R.A., COIA, L.R., RICHTER, M.P. & 5 others (1985). Oxygen tension in human tumours: *in vivo* mapping using CT-guided probes. *Radiology*, **156**, 211.

GATENBY, R.A., KESSLER, H.B., ROSENBLUM, J.S. & 4 others (1988). Oxygen distribution in squamous cell carcinoma metastases and its relationship to outcome of radiation therapy. *Int. J. Radiat. Oncol. Biol. Phys.*, **14**, 831.

GRAY, L.H., CONGER, A.D., EBERT, M., HORNSEY, S. & SCOTT, O.C.A. (1953). The concentration of oxygen dissolved in tissue at the time of irradiation as a factor in radiotherapy. *Br. J. Radiol.*, **26**, 638.

GROEBE, K. & VAUPEL, P. (1988). Evaluation of oxygen diffusion distances in human breast cancer xenografts using tumour-specific *in vivo* data: role of various mechanisms in the development of tumour hypoxia. *Int. J. Radiat. Oncol. Biol. Phys.*, **15**, 691.

GULLINO, P.M., GRANTHAM, F.H. & COURTNEY, A.H. (1967). Glucose consumption by transplanted tumours *in vivo*. *Cancer Res.*, **27**, 1031.

HILL, R.P. (1987). Ch. 10 Metastasis and Ch. 15 Cellular Basis of Radiotherapy. In *The Basic Science of Oncology*, Tannock, I.F. & Hill, R.P. (eds). pp. 160–175 and pp. 237–255. Pergamon Press: New York.

HILL, R.P. & STANLEY, J.A. (1975). The response of hypoxic B16 melanoma cells to *in vivo* treatment with chemotherapeutic agents. *Cancer Res.*, **35**, 1147.

HILL, R.P. & BUSH, R.S. (1977). A new method of determining the fraction of hypoxic cells in a transplantable murine sarcoma. *Radiat. Res.*, **70**, 141.

HORAK, E., DARLING, D.L. & TARIN, D. (1986). Analysis of organ-specific effects on metastatic tumour formation by studies *in vitro*. *J. Natl Cancer Inst.*, **76**, 913.

JÄHDE, E., GLÜSENKAMP, K.-H. & RAJEWSKY, M.F. (1989). Increased drug cytotoxicity at reduced pH counteracts cyclophosphamide resistance in cultured rat mammary carcinoma cells. *Int. J. Cancer*, **44**, 1082.

JÄHDE, E., GLÜSENKAMP, K.-H. & RAJEWSKY, M.F. (1990). Protection of cultured malignant cells from mitoxantrone cytotoxicity by low extracellular pH: a possible mechanism for chemoresistance *in vivo*. *Eur. J. Cancer*, **26**, 101.

KALLINOWSKI, F., VAUPEL, P., RUNKEL, S. & 5 others (1988). Glucose uptake, lactate release, ketone body turnover, metabolic micromilieu, and pH distributions in human breast cancer xenografts in nude rats. *Cancer Res.*, **48**, 7264.

KVANLI, A.H. (1988). Statistics. A computer integrated approach. West Publishing Company: St Paul.

LEE, A.S. (1987). Coordinated regulation of a set of genes by glucose and calcium ionophores in mammalian cells. *Trends Biochem. Sci.*, **12**, 20.

LING, V., CHAMBERS, A.F., HARRIS, J.F. & HILL, R.P. (1985). Quantitative genetic analysis of tumour progression. *Cancer Mets. Rev.*, **4**, 173.

LIOTTA, L.A. (1986). Tumour invasion and metastases – role of the extracellular matrix: rhoads memorial award lecture. *Cancer Res.*, **46**, 1.

LIOTTA, L.A., MANDLER, R., MURANO, G. & 4 others (1986). Tumour cell autocrine motility factors. *Proc. Natl Acad. Sci. USA*, **83**, 3302.

MUELLER-KLIESER, W., WALENTA, S., PASCHEN, W., KALLINOWSKI, F. & VAUPEL, P. (1988). Metabolic imaging in microregions of tumours and normal tissues with bioluminescence and photon counting. *J. Natl Cancer Inst.*, **80**, 842.

NEWELL, K.J. & TANNOCK, I.F. (1989). Reduction of intracellular pH as a possible mechanism for killing cells in acidic regions of solid tumours: effects of carbonylcyanide-3-chlorophenylhydrazone. *Cancer Res.*, **49**, 4477.

OSSOWSKI, L. & REICH, E. (1983). Antibodies to plasminogen activator inhibit human tumour metastasis. *Cell*, **35**, 611.

PELHAM, H.R.B. (1986). Speculations on the functions of the major heat shock and glucose-regulated proteins. *Cell*, **46**, 959.

POUYSSÉGUR, J., SHIU, R.P.C. & PASTAN, I. (1977). Induction of two transformation-sensitive membrane polypeptides in normal fibroblasts by a block in glycoprotein synthesis or glucose deprivation. *Cell*, **11**, 941.

POUYSSÉGUR, J., SARDET, C., FRANCHI, A., L'ALLEMAIN, G. & PARIS, S. (1984). A specific mutation abolishing $Na^+/H^+$ antiport activity in hamster fibroblasts precludes growth at neural and acidic pH. *Proc. Natl Acad. Sci. USA*, **81**, 4833.

RICE, G.C., HOY, C. & SCHIMKE, R.T. (1986). Transient hypoxia enhances the frequency of dihydrofolate reductase gene amplification in Chinese hamster ovary cells. *Proc. Natl Acad. Sci. USA*, **83**, 5978.

RICE, G.C., LING, V. & SCHIMKE, R.T. (1987). Frequencies of independent and simultaneous selection of Chinese hamster cells for methotrexate and doxorubicin (adriamycin) resistance. *Proc. Natl Acad. Sci. USA*, **84**, 9261.

SCIANDRA, J.J. & SUBJECK, J.R. (1983). The effects of glucose on protein synthesis and thermosensitivity in Chinese hamster ovary cells. *J. Biol. Chem.*, **258**, 12091.

SCIANDRA, J.J., SUBJECK, J.R. & HUGHES, C.S. (1984). Induction of glucose-regulated proteins during anaerobic exposure and of heat-shock proteins after reoxygenation. *Proc. Natl Acad. Sci. USA*, **81**, 4843.

SHEN, J., HUGHES, C., CHAO, C. & 4 others (1987). Coninduction of glucose-regulated proteins and doxorubicin resistance in Chinese hamster cells. *Proc. Natl Acad. Sci. USA*, **84**, 3278.

SHIU, R.P.C., POUYSSÉGUR, J. & PASTAN, I. (1977). Glucose depeltion accounts for the induction of two transformation-sensitivie membrane proteins in Rous sarcoma virus-transformed chick embryo fibroblasts. *Proc. Natl Acad. Sci. USA*, **74**, 3840.

SLOANE, B.F., HONN, K.V., SADLER, J.G., TURNER, W.A., KIMPSON, J.J. & TAYLOR, J.D. (1982). Cathepsin B activity in B16 melanoma cells: a possible marker for metastatic potential. *Cancer Res.*, **42**, 980.

SMITH, E., STRATFORD, I.J. & ADAMS, G.E. (1980). Cytotoxicity of Adriamycin on aerobic and hypoxic Chinese hamster V79 cells *in vitro*. *Br. J. Cancer*, **41**, 568.

SUBJECK, J.R. & SHYY, T.-T. (1986). Stress protein systems of mammalian cells. *Am. J. Physiol.*, **250** (Cell Physiol. 19), C1.

TANNOCK, I.F. (1972). Oxygen diffusion and the distribution of cellular radiosensitivity in tumours. *Br. J. Radiol.*, **45**, 515.

TANNOCK, I.F. (1982). Response of aerobic and hypoxic cells in a solid tumour to Adriamycin and cyclophosphamide and interaction of the drugs with radiation. *Cancer Res.*, **42**, 4921.

TANNOCK, I.F. (1987). Experimental chemotherapy. In *The Basic Science of Oncology*, Tannock, I.F. & Hill, R.P. (eds) pp. 308–325. Pergamon Press: New York.

TANNOCK, I.F. & ROTIN, D. (1989). Acid pH in tumours and its potential for therapeutic exploitation. *Cancer Res.*, **49**, 4373.

THOMLINSON, R.H. & GRAY, L.H. (1955). The histological structure of some human lung cancers and the possible implications for radiotherapy. *Br. J. Cancer*, **9**, 537.

TOMASOVIC, S.T. & WELCH, D.R. (1986). Heat stress proteins and experimental cancer metastasis. *Int. J. Hyperthermia*, **2**, 253.

TURNER, G.A. (1979). Increased release of tumour cells by collagenase at acid pH: possible mechanism for metastasis. *Experientia*, **35**, 1657.

VAUPEL, P., KALLINOWSKI, F. & OKUNIEFF, P. (1989). Blood flow, oxygen and nutrient supply, and metabolic microenvironment of human tumours: a review. *Cancer Res.*, **49**, 6449.

WELCH, W.J. (1987). The mammalian heat shock (or stress) response: a cellular defense mechanism. *Adv. Exp. Med. Biol.*, **225**, 287.

WHELAN, S.A. & HIGHTOWER, L.E. (1985). Differential induction of glucose-regulated and heat shock proteins: effects of pH and sulfhydryl-reducing agents on chicken embryo cells. *J. Cell. Physiol.*, **125**, 251.

WIKE-HOOLEY, J.L., HAVEMAN, J. & REINHOLD, J.S. (1984). The relevance of tumour pH to the treatment of malignant disease. *Radiother. Oncol.*, **2**, 343.

YAMORI, T., IIDA, H., TSUKAGOSHI, S. & TSURUO, T. (1988). Growth stimulating activity of lung extract on lung-colonizing colon 26 clones and its partial characterization. *Clin. Expl. Metastasis*, **6**, 131.

YOUNG, S.D. & HILL, R.P. (1986). Dynamic heterogeneity: isolation of murine tumour cell populations enriched for metastatic variants and quantification of the unstable expression of the phenotype. *Clin. Expl. Metastasis*, **4**, 153.

YOUNG, S.D., MARSHALL, R.S. & HILL, R.P. (1988). Hypoxia induces DNA overreplication and enhances metastatic potential of murine tumour cells. *Proc. Natl Acad. Sci. USA*, **85**, 9533.

YOUNG, S.D. & HILL, R.P. (1990*a*). Effects of reoxygenation on cells from hypoxic regions of solid tumours: anticancer drug sensitivity and metastatic potential. *J. Natl Cancer Inst.*, **82**, 371.

YOUNG, S.D. & HILL, R.P. (1990*b*). Effects of reoxygenation on cells from hypoxic regions of solid tumours: analysis of transplanted murine tumours for evidence of DNA overreplication. *Cancer Res.*, **50**, 5031.

ZIMMERMANN, P.V., HAWSON, G.A.T., BINT, M.H. & PARSONS, P.G. (1987). Ploidy as a prognostic determinant in surgically treated lung cancer. *Lancet*, **ii**, 530.